# From sauropsids to mammals and back: New approaches to comparative cortical development

**DOI:** 10.1002/cne.23871

**Published:** 2015-08-20

**Authors:** Juan F. Montiel, Navneet A. Vasistha, Fernando Garcia‐Moreno, Zoltán Molnár

**Affiliations:** ^1^ Medical Research Council Functional Genomics Unit, Department of Physiology, Anatomy and Genetics, University of OxfordOxfordUK; ^2^ Centre for Biomedical Research, Facultad de Medicina, Universidad Diego PortalesSantiagoChile; ^3^ Department of PhysiologyAnatomy and Genetics, University of OxfordOxfordUK; ^4^ Centre for Clinical Brain Sciences, University of EdinburghScotlandUK

**Keywords:** intermediate progenitors, progenitors, ventricular radial glia, subventricular zone, subventricular (outer) radial glia, transcriptomics, cerebral cortex, Wulst, dorsal ventricular ridge

## Abstract

Evolution of the mammalian neocortex (isocortex) has been a persisting problem in neurobiology. While recent studies have attempted to understand the evolutionary expansion of the human neocortex from rodents, similar approaches have been used to study the changes between reptiles, birds, and mammals. We review here findings from the past decades on the development, organization, and gene expression patterns in various extant species. This review aims to compare cortical cell numbers and neuronal cell types to the elaboration of progenitor populations and their proliferation in these species. Several progenitors, such as the ventricular radial glia, the subventricular intermediate progenitors, and the subventricular (outer) radial glia, have been identified but the contribution of each to cortical layers and cell types through specific lineages, their possible roles in determining brain size or cortical folding, are not yet understood. Across species, larger, more diverse progenitors relate to cortical size and cell diversity. The challenge is to relate the radial and tangential expansion of the neocortex to the changes in the proliferative compartments during mammalian evolution and with the changes in gene expression and lineages evident in various sectors of the developing brain. We also review the use of recent lineage tracing and transcriptomic approaches to revisit theories and to provide novel understanding of molecular processes involved in specification of cortical regions. J. Comp. Neurol. 524:630–645, 2016. © 2015 The Authors. The Journal of Comparative Neurology Published by Wiley Periodicals, Inc.

Understanding how the mammalian neocortex (isocortex) evolved to its present complex state is a fascinating topic for neuroscience, genetics, bioinformatics, and comparative biology. Mammals have a six‐layered neocortex, a trait that differentiates this clade from any other classes (Krubitzer and Kaas, 2005; Fig. 1). The impact of that characteristic on the emergence of associative cognitive repertoires is believed to be a key to the success of mammals. Our brains allow us to anticipate the future, create music, poetry, and to enjoy intellectual challenges that provide few practical rewards or material gains. These abilities have evolved recently (Molnár and Pollen, 2014). Studies of the changes that occurred previously as the sauropsids and mammals started to diverge may well shed light on the nature of changes responsible for these abilities. Our review examines the earliest stages of cortical development (in mammals and sauropsids) and analyzes the developmental mechanisms that evolved to produce the mammalian cerebral cortex. We review some of the most pertinent differences in brain organization and investigate the possible developmental origins of these differences to define the causes of the separate phylogenetic developments.

**Figure 1:**
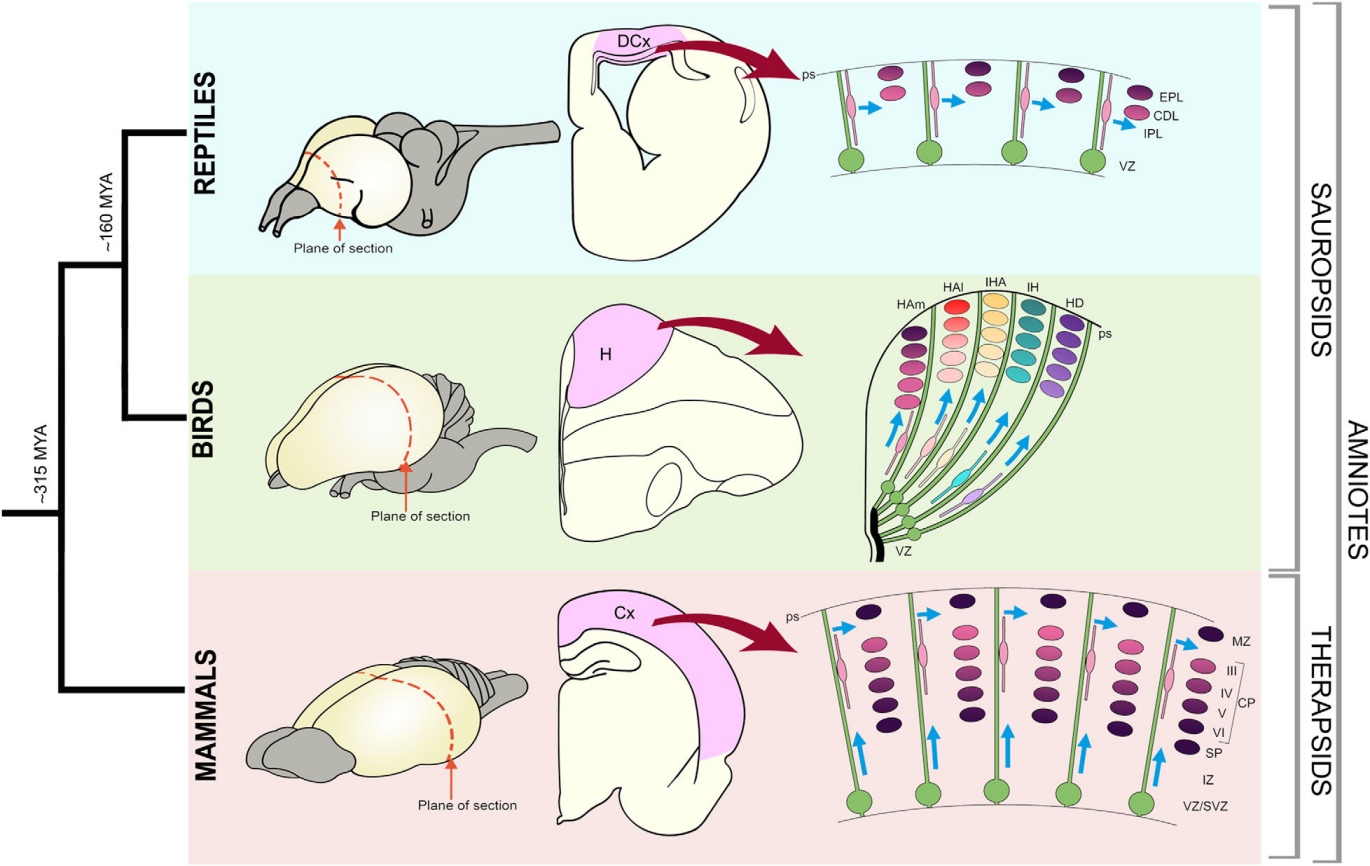
Phylogenetic relationship of mammals, birds, and reptiles. Schematic representations of brains with the plane of sections (coronal) indicated (dotted red lines). Schematic representation of sections through the reptile, bird, and mammalian brains with dorsal cortex (DCx), hyperpallium (H), and cerebral cortex (Cx) shaded in pink. The generation of the cortical neurons is schematized in the right panels. In the reptilian brain the progenitors (green) divide in the ventricular zone (VZ) and the postmytotic cells produce the cortex that is comprised of internal plexiform layer (IPL), cell dense layer (CDL), and external plexiform layer (EPL). The progenitors extend from the VZ to pial surface (ps) and generate waves of neurons that are different in different sectors. The bird hyperpallium has regional variations and several radial segments can be distinguished. A more homogeneous population of neurons of the VI layers (VI–I) are generated from progenitors of the cortex. It is postulated that the neurons of different cortical layers is generated from the same progenitors in a sequential fashion.

Neurons of the neocortex originate in the ventricular zone (VZ), a pseudostratified proliferative epithelium containing multipotent neural progenitor cells located at the deep ventricular surface of the telencephalic wall (Fig. 1). The surface adjacent to the ventricular zone is referred to as ventricular/apical, whereas the surface towards the pial surface is termed outer/basal. The progenitors adjacent to the ventricular surface are called radial (or apical) progenitors, while progenitors in the subventricular zone are termed intermediate (or basal) progenitors. A third progenitor subtype is also present basally and termed the outer radial glia (oRG), due to their abundance in the outer subventricular zone of primate cortices. Despite their nomenclature, they have also been described in the inner subventricular zone (ISVZ) in differing ratios (Hansen et al., 2010; Fietz et al., 2010; Reillo et al., 2010; Betizeau et al., 2013) and bordering the intermediate zone in mice (Shitamukai et al., 2011). Radial and oRG progenitors share similarities in gene expression and in the presence of a basal process from the pia. However, radial progenitors are also connected to the ventricular surface by their end feet and display inter‐kinetic nuclear migration, while oRG progenitors are detached from the ventricular surface and display somatic translocation. Intermediate progenitors, in contrast, are multipolar cells not connected to either the pial or ventricular surface and are different in terms of gene expression.

The increase of progenitor numbers and their cell types together with their increased cytoarchitectonic compartmentalization is potentially controlled by local and distant signals during neocortical development, resulting in changes in neurogenetic events that drive forebrain evolutionary changes (Fig. 1). We base our account on the recognition of a basic organization and embryonic plan that has been preserved across mammalian evolution. This serves as a substrate for further innovations that produced neocortical expansion (Smart et al., 2002; Dehay et al., 2015). Comparisons of circuits, analysis of cell lineage, and gene expression patterns between avian and mammalian brains reveals that cells originating from different lineages can develop convergent gene networks evolving to integrate into functional circuits (Dugas‐Ford et al., 2012; Suzuki et al., 2012; Belgard and Montiel, 2013; Belgard et al., 2013; Fournier et al., 2015)

## COMPARISONS BETWEEN FOREBRAIN ORGANIZATION IN MAMMALS AND SAUROPSIDS

The pallium of tetrapods has been subdivided into lateral, dorsal, medial, and ventral components based on cellular migratory patterns, cytoarchitecture, gene expression, and connectivity (Holmgren, 1922; Bruce and Neary, 1995; Puelles, 1995; Puelles et al., 2000; Brox et al., 2004; Moreno and González, 2007; Nomura et al., 2008). While these divisions share a common basic plan at early developmental stages, later developmental programs trigger differential gene expression, neurogenetic patterns (Tsai et al., 1981; Nomura et al., 2013), lamination (Medina and Reiner, 2000), connectivity (Aboitiz et al., 2002; Karten, 2013), and cytoarchitecture (Wang et al., 2010) for each brain compartment. Diversification of the adult brain in gene expression and morphology across vertebrate phylogeny makes comparisons difficult (Aboitiz and Montiel, 2007; Wang et al., 2011a; Belgard and Montiel, 2013). The cerebral hemispheres of vertebrates show enormous diversity, having differences in size and complexity distinct for each clade (Jarvis et al., 2005, 2013; Aboitiz and Montiel, 2007). Some remarkable examples of brain diversification in vertebrates can be found in fish, where cartilaginous fish have an expanded central nucleus (Aboitiz and Montiel, 2007), whereas teleosts have an everted brain (Butler and Hodos, 2005). Reptiles and birds have a pallial component termed the dorsal ventricular ridge (DVR), which becomes the most prominent telencephalic component in sauropsids (Aboitiz et al., 2003; Aboitiz and Montiel, 2007; Molnár, 2011), whereas mammals are characterized by the expansion of the neocortex, a dorsal pallial derivative. The brains of mammals and sauropsids have been compared for a century (Glenn Northcutt and Kaas, 1995), leading to different hypotheses of homology as different criteria were used.

**Figure 2:**
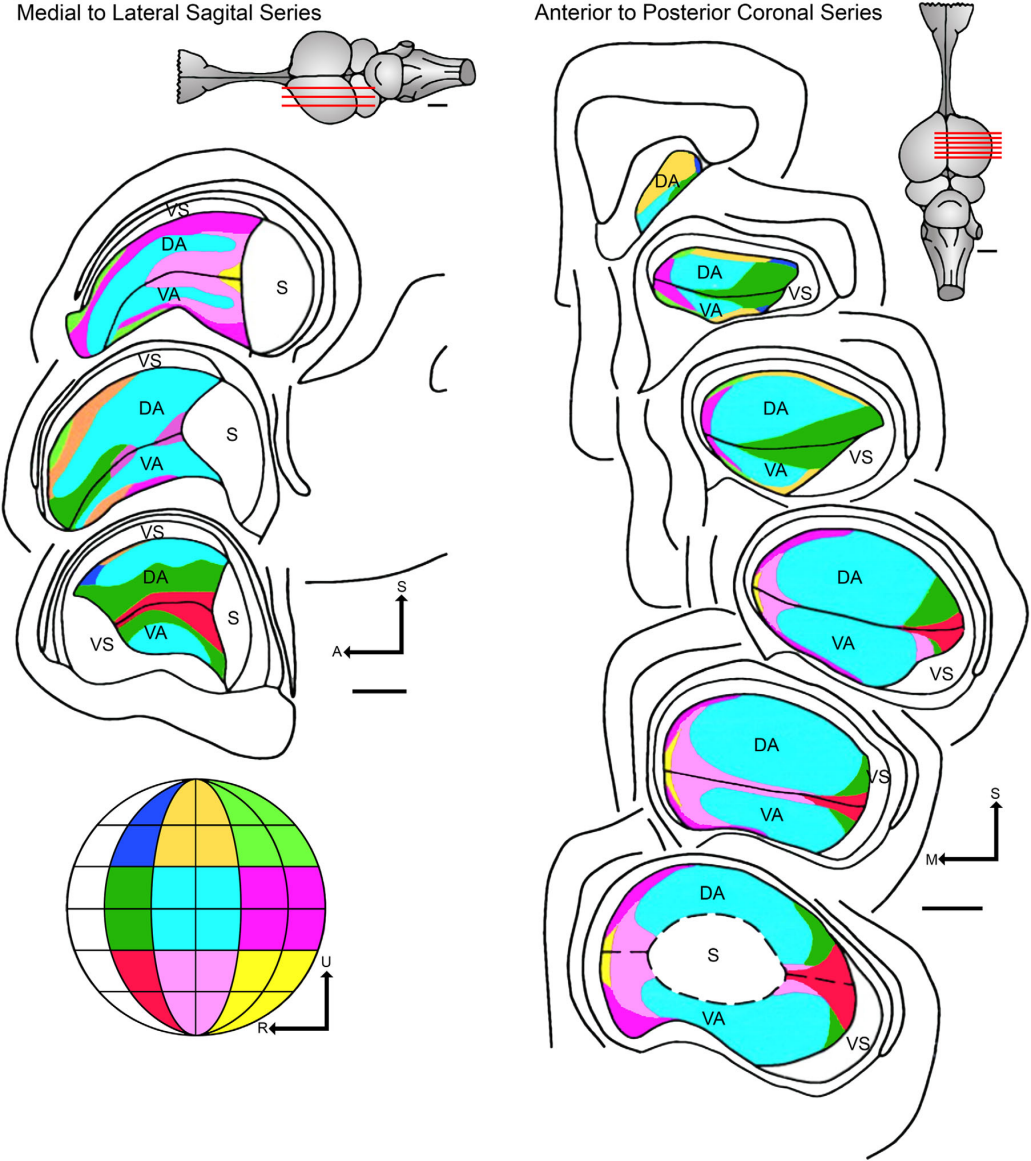
Recording from the dorsal ventricular ridge (DVR) of *Iguana* revealed a highly organized sensory representation in with highly specialized receptive field properties, similar to mammalian visual cortices. This figure has been adapted from Manger et al. (2001) and shows a color‐coded representation of the topographic organization of both the dorsal area (DA) and ventral area (VA) in both a sagittal and a coronal plane. The visual field has been divided into portions and is 60° by 60°, except those regions near the zero meridian, which have only 30° of azimuth. The central portion of the visual field (colored light blue) is the region most greatly magnified in the anterior dorsal ventricular ridge. Second to this is the adjacent region on the horizontal and vertical meridians (colored darker green). The lines across the iguana brain figurines show the approximate level of the sections shown. S: superior, M: medial, A: anterior, ADVR: anterior dorsal ventricular ridge, h: medial cortex or hippocampus, D: dorsal, dcx: dorsal cortex, lcx: lateral cortex, lat. vent: lateral ventricle, R: rostral, S: somatosensory region, U: upper, vent: ventricle, VS: visual shell. Scale bars = 1 mm.

The DVR and dorsal cortex of reptiles and the hyperpallium or Wulst of birds have been considered a homolog of the “6‐layered” mammalian neocortex (isocortex), structures that would contain equivalent circuit elements to the mammalian cerebral cortex, but distributed in a different fashion (Karten, 2013). Remarkably, recording from the DVR of iguana revealed a highly organized sensory representation with highly specialized receptive field properties, similar to mammalian visual cortices (Manger et al., 2002; Fig. 2). Similar physiological properties would not sufficiently demonstrate a common ancestral condition to prove homology, since similar properties would have evolved independently from different ancestral characters (Striedter and Northcutt, 1991). Thus, convergence must be considered as an alternative explanation. A better‐known example is the convergent evolution of vocal learning in several lineages of birds and mammals (Pfenning et al., 2015).

**Figure 3:**
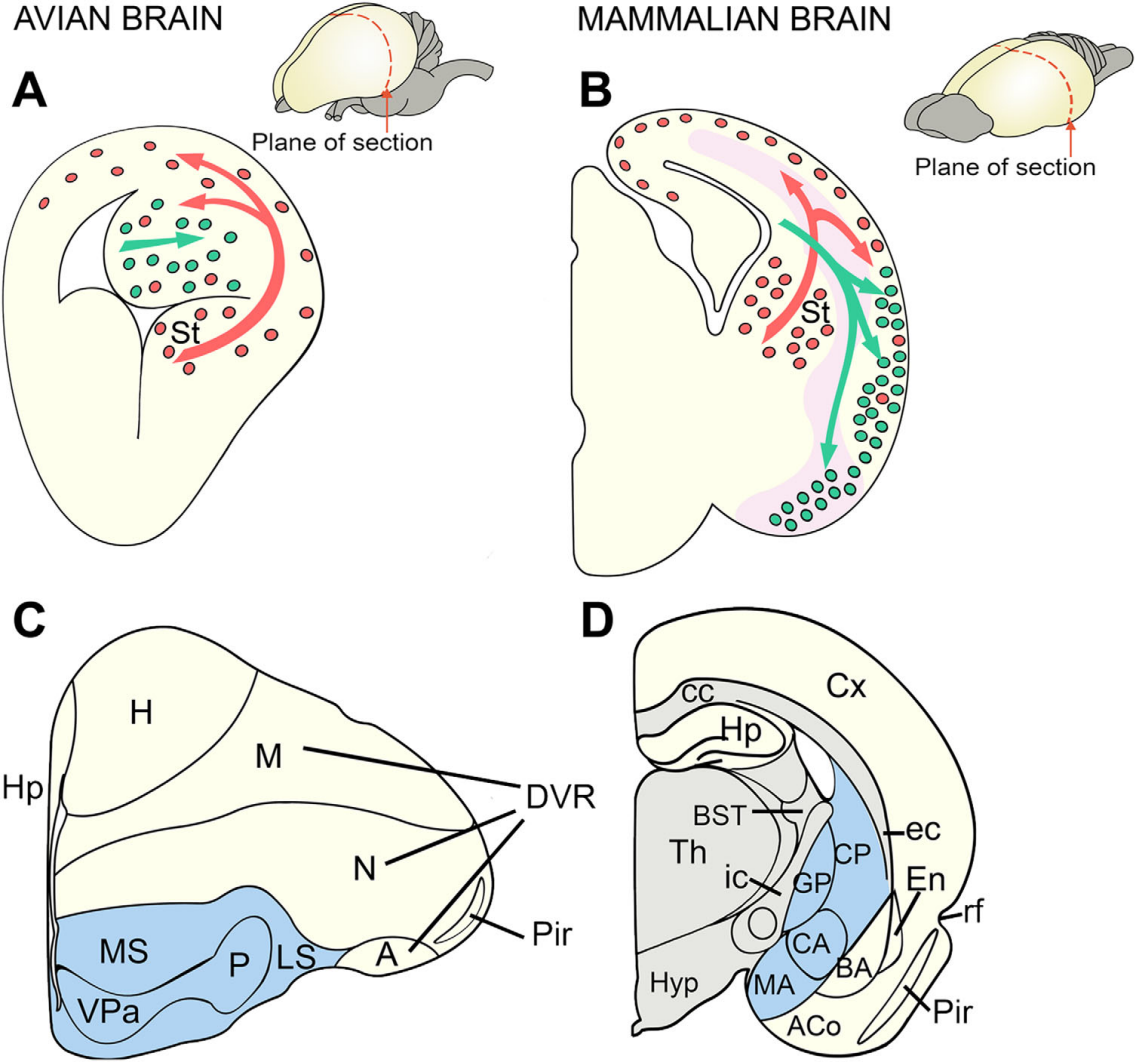
Drawings of coronal hemisections through the telencephalon in developing **(A,C)** and adult **(B,D)** sauropsids and mammals. In mammals, the Pax6 territory is indicated in pink. Inhibitory, GABAergic neuronal precursors (red dots) originate from subpallial sources and migrate tangentially into the pallium in both mammals and sauropsids. Excitatory, pyramidal‐type neuronal precursors (green dots) of the lateral migratory stream traverse the Pax6 territory to reach the lateral pallial regions in mammals but remain in situ within the DVR in sauropsids. Despite its extensive target area, the lateral migratory stream is considered a subset of the radially migrating pallial neurons. Subpallial components in the adult telencephalon are indicated in blue (C,D). Additional radial migration also occurs. Reproduced from Molnár and Butler (2002) .

The topographic position of the neocortex and the conserved expression of selected homeobox genes in the embryonic telencephalon strongly support homology of the avian hyperpallium and the reptilian dorsal pallium, hodological evidence suggests a similarity between the sensory inputs to the avian DVR and the mammalian lateral neocortex, sustaining a long‐standing debate about neocortical origins. Thus, the “thorniest questions of comparative neurobiology” (Northcutt, 2003) are whether the hyperpallium and the neocortex are homologous and whether these mammalian and avian structures contain similar circuits, but arranged in a different manner (Jarvis et al., 2013; Karten, 2013; Montiel and Molnár, 2013). The definition of homology in developmental terms would require that the two structures develop from the same sector of the developing pallium but that different developmental migratory patterns and inductive signals would drastically transform the original pallial plan (Fig. 3). Candidate homologous structures must be traceable back to a common ancestor under multiple and unbiased comparative criteria.

## ORGANIZATION OF THE AVIAN PALLIUM

**Figure 4:**
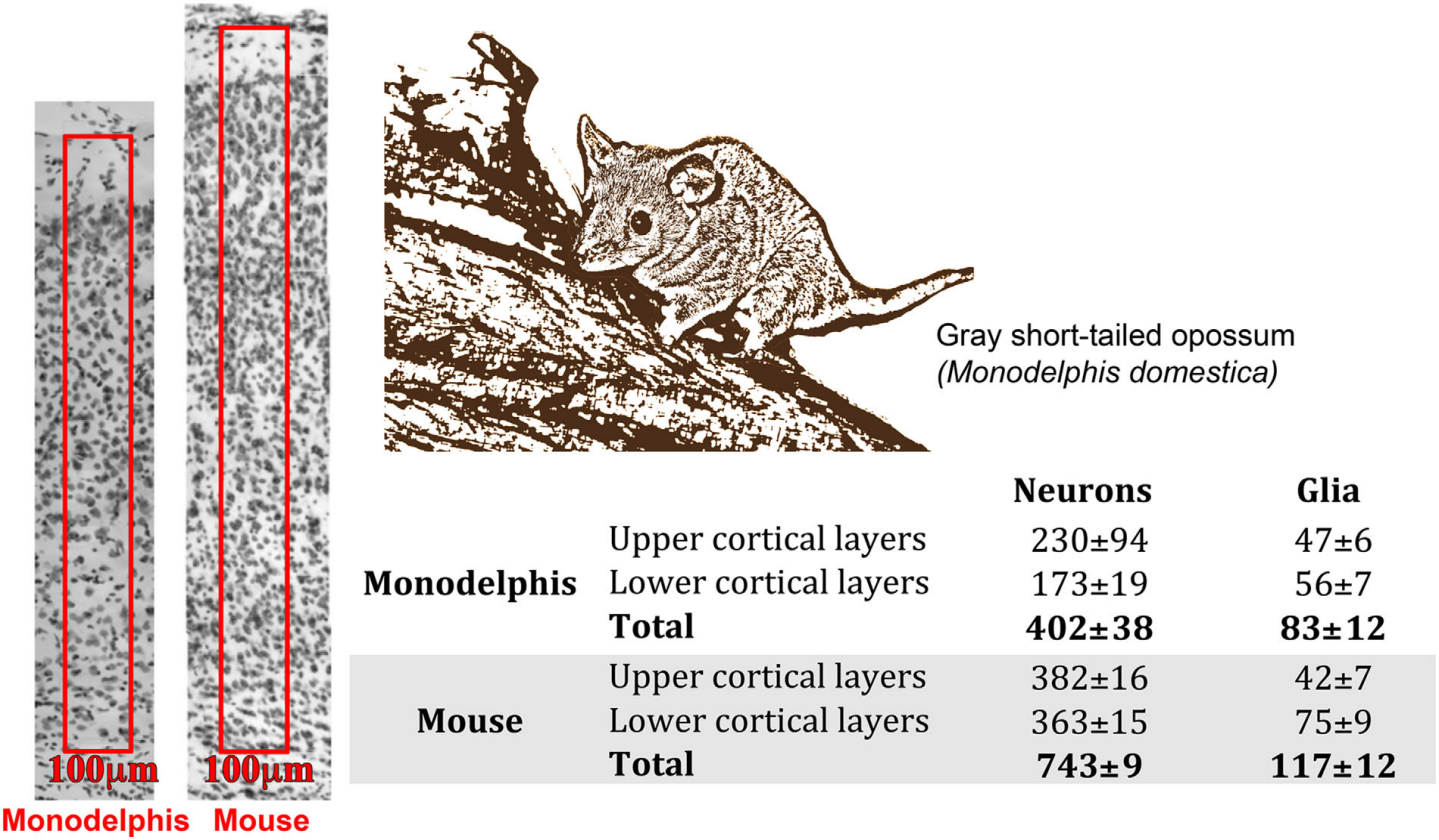
Quantification of the number of neurons in the primary somatosensory cortex of the adult mouse and *Monodelphis domestica* (picture) in a cresyl violet‐stained coronal section (Cheung et al., 2010). An arbitrary “unit column” (a 100‐μm wide, 40‐μm thick region spanning from layer 1 to 6) was marked in the primary somatosensory area (boxed areas in left panels). The number of neurons was quantified in each layer and expressed as mean ± SEM in the table. The mean number of neurons present in each cortical layer, showing that the number of neurons in a unit column is much lower in opossum as in mouse, whereas the glial numbers were less different. The number of neurons were reduced both in the upper and lower layers.

The hyperpallium is organized differently from the mammalian cortex in that it is devoid of layers. Cell groups are instead organized into nuclear‐like regions and are considered by some as a pseudolayered (columnar) organization (Medina and Reiner, 2000; Wang et al., 2010; Jarvis et al., 2013; Karten, 2013). Further, the hyperpallial layers are generally arranged parallel to the orientation of radial glial fibers, in striking contrast to the mammalian neocortical layers, which develop perpendicular to the orientation of radial glial fibers (as shown in Figs. 1 and 4). However, these “columns” are very different from those of the mammalian cortex; the arrangement of radial glia implies that neurons of each radial column of the hyperpallium must be born in separate, adjacent regions of neuroepithelium, from where they migrate toward the pial surface. Therefore, these nuclei or layers originate from different sectors of the telencephalic neuroepithelium. Remarkably, the clear‐cut inside‐first outside‐last development of the dorsal pallium has no counterpart in the avian embryonic brains (Tsai et al., 1981). With some exceptions (Striedter and Keefer, 2000), neuroblasts lack the ability to advance through older settled neurons. Consequently, early‐born neurons in the adult pallium occupy outer positions of the telencephalon (closer to the meninges), whereas late‐born neurons locate inner pallial zones (closer to the ventricle). In the developing hyperpallium of chickens, however, a clear pattern cannot be defined because early‐, mid‐, and late‐born neurons intermingle and show an apparently random distribution (Striedter and Keefer, 2000). In contrast, the pallium in quail develops roughly in an outside‐first inside‐last fashion; the cells born at early stages are distributed in both superficial and deep zones of the pallium, whereas those born at later stages are located specifically deeply in the pallium (Nomura et al., 2008). However, an evident and well‐organized inside‐out pattern can be seen only in mammalian cortex (Fig. 1).

The hyperpallium is, therefore, considered to be a nonlayered structure even though its radial columns are interconnected (Medina and Reiner, 2000; Medina and Abellán, 2009; Karten, 2013). An interesting feature of avian pallial development is the existence of DVR isochronic clusters of neurons (Striedter and Keefer, 2000). Accordingly, neurons generated concurrently in the lateral and ventral parts of the pallium tend to aggregate and form groups or clusters of cells in the DVR. This unexpected feature of the ventrolateral pallium, demonstrated with 5‐bromo‐2‐deoxyridine (BrdU), with no known counterpart in mammalian pallial development, generated considerable interest since it showed characteristics resembling the development of the striosomal compartment of the striatum (subpallial in origin) in mouse (Brand and Rakic, 1979). However, the existence of isochronic clusters in avian pallial development has been recently shown to be an artifact (Rowell and Ragsdale, 2012), who assign the appearance of isochronic DVR clusters to BrdU toxicity. Previous studies (Duque and Rakic, 2011) have undoubtedly shown the toxicity of BrdU, a commonly used reagent in birthdating studies. Birthdating in chick embryos could be reliably carried out by using lower concentrations of BrdU or other thymidine analogs (5‐chloro‐2‐deoxyuridine, CldU, and iodo‐2‐deoxyuridine, IdU), and this failed to show that cells born at the same time show a tendency to aggregate.

## SUBVENTRICULAR ZONE IN SAUROPSIDS AND MAMMALS

The structure and function of a given brain area is determined during ontogeny by the regulation of cell proliferation and migration (Cheung et al., 2010; Lui et al., 2011; Borrell and Calegari, 2014; Florio and Huttner, 2014; Taverna et al., 2014). Recent investigations have tried to establish links between the organization of the germinal zones and telencephalic structures in various species.

Reptiles possess a cortex with a sparse density of neurons at all levels of the cortex relative to mammalian values (Cheung et al., 2010). These reptilian cells are mostly comparable to infragranular early‐generated neocortical neuronal populations, mammalian layers 5 and 6 (Reiner, 1991, 2000). In our neurodevelopmental comparative studies of the turtle we found that in the telencephalon most neuronal divisions occur in the ventricular zone (VZ) of the lateral ventricle and in abventricular positions at various distances from the VZ (Abdel‐Mannan et al., 2008; Cheung et al., 2010). However, divisions outside of the VZ are infrequent and scattered in the turtle dorsal cortex, suggesting the absence or very rudimentary existence of an SVZ (Cheung et al., 2010). In chicken we described an organized SVZ for the basal ganglia, the nidopallium, and mesopallium, but found that it was absent in the hyperpallium (Cheung et al., 2010). These data suggest that the expansion and organization of the SVZ into the dorsal pallial germinative zones played a crucial role in the enlargement of the number of pallial neurons during early mammalian evolution. Comparisons of VZ and SVZ transcriptomes in the pallial and subpallial divisions will prove useful for identifying commonalities and differences among amniotes.

Although marsupials have a 6‐layered dorsal cerebral cortex similar to that of other mammals, our comparisons of cortical neuron numbers in an arbitrarily selected unit column revealed that adult South American gray short‐tailed opossums and tammar wallabies possess just half of the cerebral cortical neurons in these unit columns compared with the mouse (Cheung et al., 2010; Fig. 4). The neuronal numbers are reduced in both upper and lower cortical layers in the opossum compared to mouse, whereas the glial cell numbers are more comparable. When we examined the basic patterns of the cell divisions in the ventricular and subventricular zones we found that the basic pattern was conserved in opossum, tammar wallaby, and mouse (Cheung et al., 2010). However, the emergence of a distinctive band of dividing cells in the SVZ occurs relatively later in relation to overall brain maturation in the development of the opossum and the tammar wallaby than in mouse or rat. Interestingly, the comparisons of selected genes with known cortical VZ and SVZ expression in the mouse and opossum revealed very similar expression patterns. Even the SVZ‐specific vascular patterning was apparent (Stubbs et al., 2009; Cheung et al., 2010). These observations suggest that the smaller number of cortical neurons in the radial domain is associated with the reduction and late appearance of the SVZ in marsupials. It also suggests that the cortical SVZ and the intermediate SVZ progenitors have been conserved across all mammals that have been studied (Molnár et al., 2006).

## IS THERE AN SVZ WITH INTERMEDIATE PROGENITORS IN THE SAUROPSID HYPERPALLIUM?

In comparison with mammals, the rudimentary 3‐layered reptilian cortices lack an SVZ despite the presence of a small population of scattered abventricular mitoses (Martínez‐Cerdeño et al., 2006; Cheung et al., 2007) and expression of *Tbr2* (Clinton et al., 2014). Progenitor cells in the proliferative dorsal pallial compartment of reptiles cycle more slowly than mammalian progenitors that have a corresponding location (Nomura et al., 2013). Avian brains, in contrast to reptiles, have a substantial abventricular mitotic zone in the subpallium and the lateroventral pallium that can be considered a subventricular zone. The presence of an SVZ in the dorsal pallium of birds, however, is still debated (Striedter and Keefer, 2000; Cheung et al., 2010; and see below).

According to the definition proposed by the Boulder Committee Report (Angevine et al., 1970), the SVZ is defined as: “located at the junction of the ventricular and intermediate zone. The initial cellular occupants come into position soon after the intermediate zone has begun to form. Subventricular cells are small and round or oval. They are distinguished from the young neurons of the intermediate zone by their proliferative activity and, unlike ventricular cells, they remain stable in position, without a to‐and‐from‐nuclear displacement, during the mitotic cycle.”

According to this definition, it can be argued that many species of birds (such as chickens and quails) do not possess a well‐defined SVZ. This is not due to the absence of subventricular and scattered abventricular mitoses (which are regularly observed in the hyper, meso, and nido‐pallium), but due to absence of a “zone”‐like clustering of these mitoses (Cheung et al., 2007). However, some species (such as parakeets and zebra finches) do show aggregation of abventricular mitoses as a thin band above the VZ (Charvet et al., 2009). However, the proportion of abventricular mitoses in the hyperpallium (proposed to be homologous to the dorsal cortex of mammals (Molnár and Butler, 2002)) is negligible compared to the proportions observed in the meso and nido‐pallium (Cheung et al., 2007). Even more relevant is that chickens and quails belong to a basal avian order (Galliformes) and zebra finch and parakeets to evolutionarily distant avian groups, the songbirds (order Passeriformes and Psittaciformes, respectively) (Zelenitsky et al., 2011). Thus, abventricular mitoses that occur in a well‐defined zone would be a derived evolutionary change occurring in these late avian orders. Despite this difference in alignment of abventricular mitoses, it has been shown that developing chick brains do express *Tbr2*, a marker for basal progenitors in mammalian cortices (Englund, 2005; Suzuki et al., 2012). These cells do not consistently proliferate in an abventricular zone and we do not observe alignment of abventricular mitoses in reptiles in any sector of the pallium (Nomura et al., 2013).

## DOES THE ISOCORTEX OF ALL EUTHERIAN MAMMALS DEVELOP FROM A GERMINAL ZONE WITH SVZ?

A similar problem also exists in identifying an SVZ in metatherian mammals such as the gray short‐tailed opossum (*Monodelphis domestica*). *M. domestica*, like all marsupials, shows altricial development, being born when immature and migrating to the teats and clinging to them for a significant part of their postnatal development (Keyte and Smith, 2012). Marsupial cortices display a hexalaminar arrangement similar to that seen in eutherian mammals (Gray, 1924) leading to a debate whether the presence of an SVZ predates the eutherian–metatherian split. Few studies exist that have analyzed neurogenesis in Methatherian cortex in detail. Accounts for *Monodelphis* differ. Puzzolo and Malamacci (2010) argue against the presence of a subventricular zone (basal progenitor compartment) based on pH3, Tbr2, and BrdU immunohistochemistry. In this they differ from the conclusions of Cheung et al. (2010), who used pH3 immunostaining to show that an aggregation of abventricular mitoses exists at P16. This, however, does not answer the presence of a significant population of Tbr2+ve cells basal to the VZ during the period of neurogenesis (P1–P20) that fails to colocalize with pH3 immunoreactivity. Alternate explanations for the presence of Tbr2+ cells can be found; e.g., one can postulate a nonproliferating population of Tbr2+ cells in the abventricular zone of sauropsids that would be similar to those observed in *Monodelphis,* probably representing an ancestral condition in amniotes (Nomura et al., 2013). Cheung et al. (2010) also demonstrated the existence of SVZ divisions in the tammar wallaby, but again at a much later period than expected. Interestingly, both *Monodelphis* and tammar wallaby show a low number of neurons within an arbitrary unit column compared to mouse (Cheung et al., 2010).

It is well known that the SVZ (and the lower intermediate zone) serves as a route for migration of GABAergic interneurons in the neocortex of rodents (Del Rio et al., 1992). It has also been shown that Tbr2 regulates interneuronal migrations into the SVZ by regulating the expression of Cxcl12 (Sessa et al., 2010). In keeping with these observations, Puzzolo and Mallamaci (2010) show that GABA+ and GAD+ cells migrate into the SVZ in *Monodelphis*. Thus, it may be said that although a sparse zone of abventricular mitoses exists in birds and marsupials, they do not colocalize with known intermediate progenitor cell markers of higher mammals. This conundrum can be solved if one considers that reactivity of Tbr2 (or other markers) is not a sine‐qua‐non for the SVZ. Instead, abventricular mitoses in these animals might have a separate identity from that of rodents and higher mammals that either has been lost or has coalesced with the intermediate progenitor cell (IPC) marker (Tbr2) during the evolution of the neocortex. A comparative large‐scale gene expression study could well serve to outline the differences between the abventricular divisions in these species for the SVZ and VZ compartments.

At the other end of the mammalian spectrum, species such as ferrets, marmosets, macaques, and even gyrencephalic rodents such as the agouti (*Dasyprocta agouti*) and *Capybara* show that a further increase in the neurogenic potential can be attained by a elaboration of the subventricular zone (Reillo et al., 2011; García‐Moreno et al., 2012; Kelava et al., 2012). These animals show a compartmentalization of the SVZ roughly around mid‐gestation into an inner and outer compartment termed the ISVZ and OSVZ bisected by the inner fiber layer (IFL) of unknown composition and origin (Smart et al., 2002). Initial studies have suggested that the ISVZ consists mainly of IPCs generated from the underlying radial glia (RGCs); in contrast, the OSVZ consists of a mixture of IPCs and a recently described progenitor; the outer radial glia (oRG) that is similar in gene expression and function to the RGCs whose nuclei found in the VZ (Hansen et al., 2010; Fietz and Huttner, 2011; Reillo et al., 2011). However, more recent studies in ferret (Reillo and Borrell, 2012) and macaque (Betizeau et al., 2013) have shown that the cellular composition of ISVZ and OSVZ is very similar and both contain oRG and IPCs than was previously appreciated. This is also in agreement with the finding that gene expression profiles of ISVZ and OSVZ in the human developing neocortex are closely related to each other (Fietz et al., 2012; Miller et al., 2014).

The IPCs contained in the OSVZ may originate from both the VZ and the OSVZ through the asymmetric division of oRG progenitors. A cursory examination of the cell numbers in the IPC versus oRG in the OSVZ in marmoset brains has shown an excess of IPCs relative to oRG cells. This may indicate a dominant role of IPCs in the generation of neurons in species with an OSVZ. Thus, it seems more likely that an elaboration of the germinal zones (brought about by the cytoarchitectonic separation of the SVZ into ISVZ and OSVZ) is a more conservative indicator of a larger‐brained mammal than the presence of a specific progenitor; namely, the oRG cells. This is further supported by the observation that mouse brains that do not possess an OSVZ contain a small percentage of oRG cells in the upper IZ (Shitamukai et al., 2011; Wang et al., 2011b). It is important to note here that unlike IPCs, oRG have never been observed in any nonmammalian species in any of the species studied so far. This would suggest that oRG in the pallium might be considered a mammalian‐specific trait but further comparative studies are needed.

Recent lineage tracing from our group using the *Tbr2^Cre^* mouse line shows that cortical neuronal progenies from Tbr2+ IPCs occupy all cortical layers (Vasistha et al., 2014). Importantly, 25.44 ± 2.84% of all infragranular layer cells are derived from the IPC lineage with the numbers going up to 35.55 ± 3.24% for the supragranular layers. This suggests that the difference between the contribution made by IPCs towards the infragranular and supragranular layers is not as discordant as previously thought based on gene expression (Tarabykin et al., 2001). On comparing the lineage tracing data with those of Tbr2 conditional knockout (cKO) animals, it is interesting to note that only layers 4 and 6 show any appreciable difference in number of cells in the cKO (Vasistha et al., 2014). This suggests the role of other compensatory genes in the generation of neurons for other layers. The lineage tracing study of Vasistha et al. (2014) also shows that IPCs generate only excitatory neuronal progenies and no astrocytes or oligodendrocytes. The glutamatergic specific generation of neurons by Tbr2+ progenitors is similar in the subependymal zone of the adult mouse where they generate olfactory bulb interneurons (Brill et al., 2009).

## TEMPORAL VERSUS SPATIAL MODELS OF PALLIAL PROGENITORS FATE RESTRICTIONS

**Figure 5:**
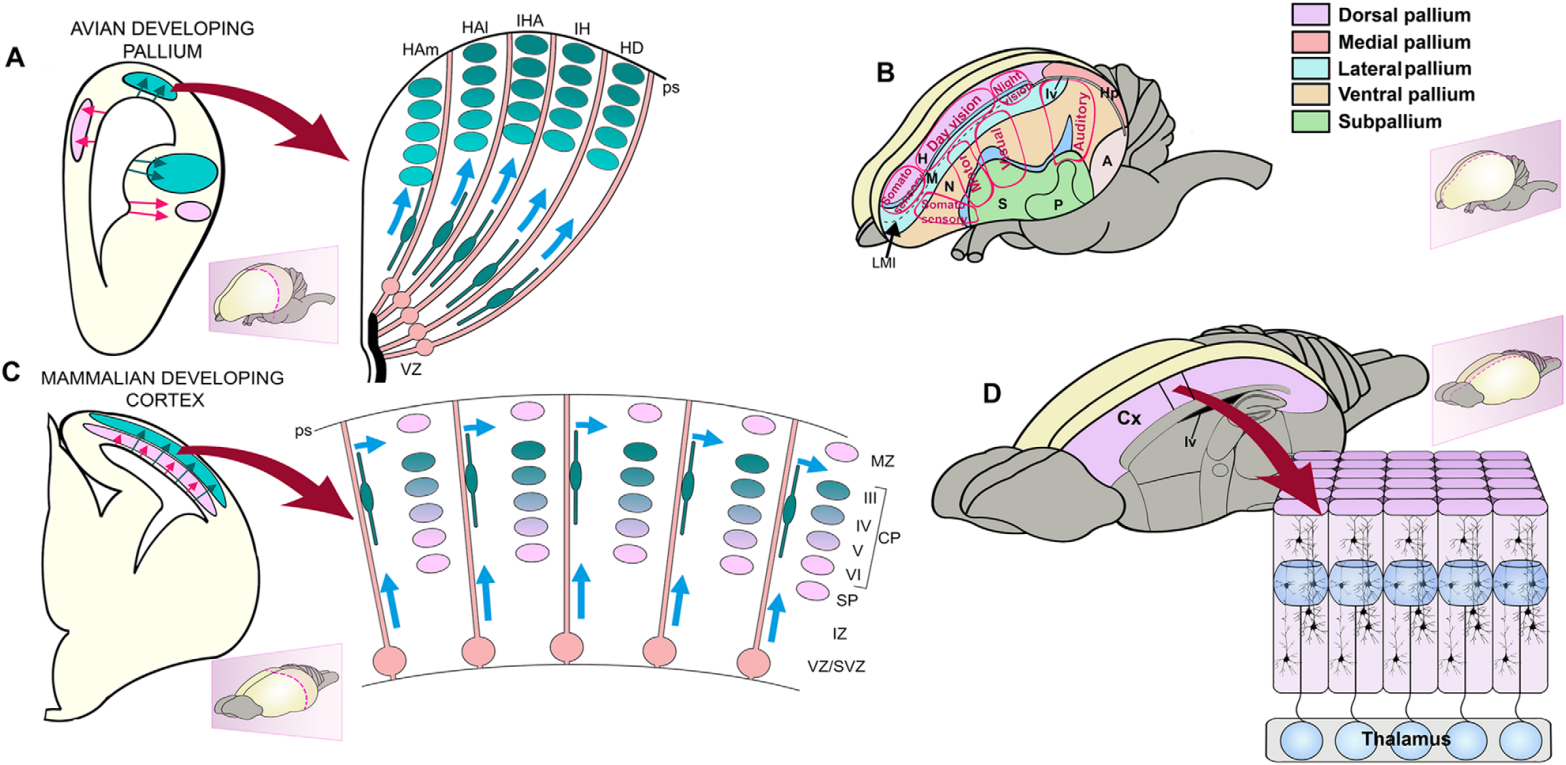
The production and assembly of neuronal circuits are different in the avian and mammalian brain. In the avian pallium, different sectors contain different fate‐restricted progenitors that produce different elements that combine perpendicular to the radial trajectories. In mammals the diverse elements of the functional columns are produced within the same sector of cortical neuroepithelium from sequential neurogenesis from radial glia progenitors and assemble along the radial trajectories. In avian brains the neurons that assemble into functional units organized perpendicular to boundaries between pallial sectors (somatosensory, motor, auditory, night and day vision) that extended through different sectors of both dorsal and ventral avian pallium. In mammalian cortex the distinguishable radial columns are formed parallel to the orientation of the radial glia and not perpendicular to them. Mammalian columns extend across the layers of the cerebral cortex perpendicular to the pial surface. The neurons are arranged within the same radial pallial sector that gave rise to them in mammals. The elements of the coactivated neuronal ensembles have very different developmental origin in mammals and in birds.

Since the identification of the characteristic in‐side‐first out‐side‐last mammalian cortical neurogenetic gradient it has been assumed that all progenitors in a given brain area are similar in their molecular and kinetic features. They would generate most cortical cell types in a sequence, as they become progressively fate restricted. However, increasing evidence shows several types of progenitor pools in telencephalic germinative zones (Marín and Müller, 2014) and even different progenitor types residing in the same pool. The generation of different cell types across the pallium relies on temporal and spatial distinctions. Recent findings in chick development suggest that the temporal sequence of generation (temporal code) of cortical neurons was already inherent in the stem amniote (Suzuki et al., 2012; Fig. 5). However, this temporal code has not been selected across avian evolution, and in the specialization of different parts of the pallial germinal zone a spatial code became dominant (Nomura et al., 2008; Suzuki et al., 2012). According to specific markers of mammalian cortical layers and GFP tracing in chick by in ovo electroporation, Suzuki et al. (2012) demonstrated that medially located dorsal pallial progenitors generate neurons that express selected markers of deep‐layers, whereas laterally located dorsal pallial progenitors are restricted to generate neurons later in pallial neurogenesis that will express selected upper‐layer markers. Currently, it is not known which of the two neurogenic codes applies to development in reptilian species. Moreover, it is not clear whether the reptilian and avian dorsal pallial regions with similar gene expressions to the upper and lower layers can be considered homologous to mammalian cerebral cortical layers. Would these comparisons stand the test of studies based on the whole transcriptome?

## EXPLORING BRAIN EVOLUTION FROM TRANSCRIPTOME DATABASES

The entire set of RNA molecules, including mRNA, rRNA, tRNA, and other noncoding RNA found at cellular, tissue, organ, or whole body levels constitute the transcriptome. Transcriptomes are spatially organized within regions of the brain and even within single cells (nuclear, cytoplasmic synaptic, macromolecular complexes). Transcriptomes can also be temporally regulated, can change during functional states, or can exhibit circadian dynamics; due to which only a subset of the transcriptome is usually measured at any one time. Nevertheless, transcriptomes display conserved features based on which developmental and adult nervous system compartments can be potentially classified by their transcriptomic constituents. Transcriptomic comparison using unbiased in silico methods can be used to derive a set of genes that can be categorized and correlated accordingly in order to define several features, for example: expression‐enrichment, coexpression, specificity, functional‐enrichments, interactions, ancestry, etc.

During the development of the central nervous system, a progressive transcriptomic differentiation is observed for different segments of the neural tube. Hundreds of genes are differentially expressed between the anterior and posterior poles of the neural tube and correlate with different neurodevelopmental abnormalities with segmental preferences (Krupp et al., 2012). In the brain, transcriptomes from the whole cerebral cortex, and developing neocortical brain compartments (ventricular zone, subventricular zone, and cortical plate, CP), exhibit different sets of transcripts. Developmental compartments have been shown to express specific gene‐signatures (Ayoub et al., 2011; Fietz et al., 2012) differing across ontogeny. Thus, a cohort of active transcripts during development and in adult cortical compartments can be differentiated at the level of gene expression, splicing, and RNA editing (Dillman et al., 2013). Differential set of transcripts are also expressed across phylogeny: thus, neocortical common and species‐specific gene signatures can be found for closely related species like monkey and human, and across more distant species like mouse and human (Ayoub et al., 2011; Hou et al., 2012). High‐throughput analysis of all active transcripts can be a powerful criterion for comparisons across brain sectors and for defining homologies (Belgard et al., 2013; Table 1).

**Table 1:** Overview of Brain Comparative Transcriptomic and Epigenomic Studies in Amniotes

Species	Analysis	Conclusions	Reference
Mouse (*M. musculus*), Human (*H .sapiens*)	RNA microarray from GEO datasets	Identify human and mouse specific modules and also correlation of human modules with neurodegeneration	Miller et al., 2010
Chicken (*G. gallus*), Opossum (*M. domestica*), Mouse (*M. musculus*)	Genome‐wide long‐non coding RNA (lncRNA)	Identified 4 lncRNA conserved in distant amniotes	Chodroff et al., 2010
Human (*H. sapiens*), Chimpanzee (*P. troglodytes*), Macaque (*M. mulatta*)	RNA microarray and RNA‐sequencing	Synaptic related genes are extended in human development. Identify human specific gene expression modules described.	Liu et al., 2012
Human (*H. sapiens*), Chimpanzee (*P. troglodytes*), Macaque (*M. mulatta*)	RNA sequencing	Human specific co‐expression modules identified. These correlate with neuronal maturation genes and also neuropsychiatric disease linked genes.	Konopka et al., 2012
Chicken (*G. gallus*), Mouse (*M. musculus*)	RNA‐sequencing	Some regions with common developmental origins do not display transcriptomic similarity.	Belgard et al., 2013
Human (*H. sapiens*), Chimpanzee (*P. troglodytes*), Macaque (*M. mulatta*)	RNA‐sequencing	Few significant RNA‐editing level changes between human, chimpanzee and macaque PFC and cerebellum.	Li et al., 2013
Zebra finch (*T.guttataI*), Budgerigars (*M. undulates*), Anna's hummingbirds (*C. anna*), Human (*H. sapiens*), Macaque (*M. mulatta*)	RNA microarray	Gene expression correlation between vocal‐learning songbirds and human cortex and striatum but inverse relation with amygdala.	Pfenning et al., 2014
Human (*H. sapiens*), Macaque (*M. mulatta*)	RNA‐sequencing	Conserved expression of lncRNA between macaque and human PFC.	He et al., 2014
Human (*H. sapiens*), Macaque (*M. mulatta*), Mouse (*M. musculus*)	Epigenetic profiling by ChIP‐seq for H3K27ac and H3K4me2 associated sequences.	Identified several genes and modules showing human specific gain of promoter or enhancer regions.	Reilly et al., 2015

High‐throughput RNA sequencing and microarrays techniques have made it possible to analyze large transcriptomic networks to explore evolutionary changes acting on existing developmental apparatus to create the observed diversity of adult brain forms described previously. At several brain developmental stages, cortical proliferative compartments have been compared between human and mouse, exhibiting a pattern of similarity between progenitor and derived postmitotic compartments (Fietz et al., 2012). This is probably associated with the radial expansion and more elaborate development of the cortical program in humans. Fietz et al. (2012) demonstrated that the transcriptome of the mouse SVZ is more closely related to that of the CP than to the transcriptome of the human SVZ, which is more like that of the human VZ. The telencephalic VZ can be further subdivided in the tangential domain into several specific germinal pools. Four main subdivisions in the pallial proliferative domains can be distinguished: the medial, dorsal, lateral, and ventral pallia and each of them gives rise radially to different nuclei or cortical structures depending on the taxa. Thus, a transcriptomic analysis involving these divisions is of major interest in order to identify early different genetic programs and to make comparisons across species. Subdivisions of the germinal subpallium are still not fully established. It has been proposed that a number of subdivisions up to 17 subregions can be differentiated at the transcriptomic level in mice (Flames et al., 2007).

**Figure 6:**
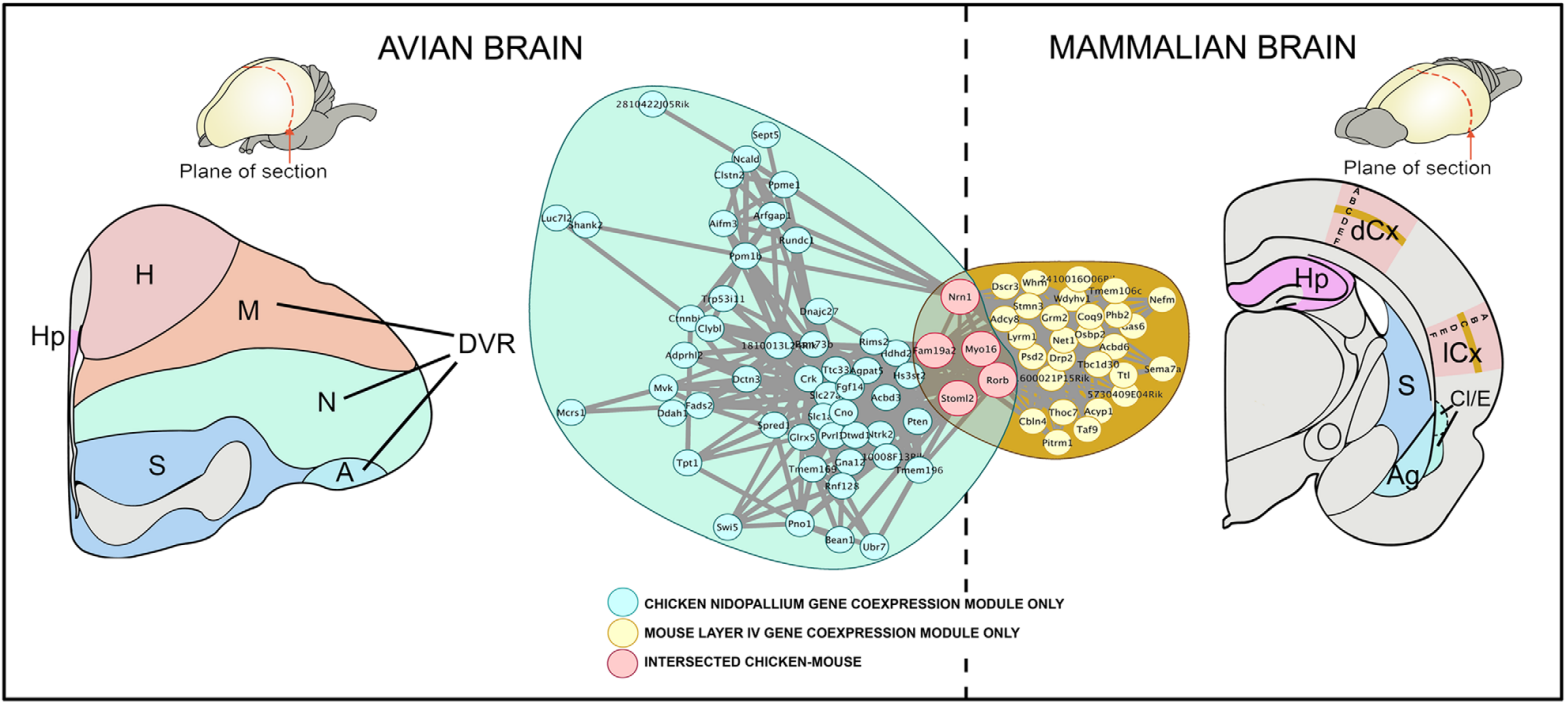
Convergent gene coexpression modules marking chicken nidopallium and mouse layer IV. We explored which genes are actively transcribed in the regions of controversial ancestry in a representative bird (chicken) and mammal (mouse) at adult stages (Belgard et al., 2013). This study conducted four analyses comparing the expression patterns of their 5,130 most highly expressed one‐to‐one orthologous genes that considered global patterns of expression specificity, strong gene markers, and coexpression networks. Expression correlations between genes in significantly overlapping gene coexpression modules expressed in chicken nidopallium and mouse layer IV (Bonferroni‐corrected *P* = 4.6 × 10^‐3^, hypergeometric test). Nidopallium and neocortical layer IV are generally considered by the community of comparative neurobiologists functionally analogous and hodologically similar to ventral and dorsal pallial derivatives. The nidopallium and layer IV showed weak but significant convergence in gene expression despite their very different developmental trajectories and agrees with previous hodological and functional. Figure modified from Belgard et al. (2013).

Our group has analyzed the gene expression associated with cells in the subplate throughout different developmental and postnatal stages. Subplate cells are some of the earliest generated neurons in cortex (Rakic and Kostovic, 1990; Hoerder‐Suabedissen et al., 2009; Oeschger et al., 2012; Hoerder‐Suabedissen, 2013). These studies strengthen the hypothesis that an embryonic subplate was present in the ancestors of mammals and that additional cellular populations evolved as cortical development and connectivity became more complex (Montiel et al., 2011; Wang et al., 2011a). In the human cortex we observed that subplate subpopulations show increased compartmentalization and that these segregate into sublayers (Hoerder‐Suabedissen and Molnár, 2015). In the adult brain, Belgard et al. (2011) produced the first transcriptomic atlas of the mouse cortical layers and provided a transcriptomic comparative gene expression analysis to obtain expression profiles of 5,130 highly transcribed genes (Belgard et al., 2013) in a set of structures that develop from one of four sectors of the pallium (medial, dorsal, lateral, and ventral) in the adult mouse and chicken brain. This recent large‐scale transcriptomic analysis reveals expression similarity across chicken and mouse between homologous telencephalic structures such as striatum and hippocampus, but a mostly divergent pattern in other telencephalic compartments (Belgard et al., 2013; Fig. 6). Belgard et al. (2013) found that structures that share a developmental origin but show functional divergence between birds and mammals do not exhibit greater similarity in transcription profiles than developmentally unrelated but functionally equivalent structures. However, regions such as the striatum and hippocampus, which share homology and function between birds and mammals, displayed conservation among groups of coexpressed and marker genes (Belgard et al., 2013). Our comparative transcriptomic analysis allowed us to critically evaluate recent studies of the avian brain that compared a relatively small number of genes (Dugas‐Ford et al., 2012; Suzuki et al., 2012; Montiel and Molnár, 2013). Indeed, our data suggest there are genes that, if selected, could be used to support many different relationships (Montiel and Molnár, 2013; Fig. 5). A correct interpretation of these results is that different patterns of similarity would emerge when a few selected genes are compared at adult stages; nevertheless, many of these observations are not significantly supported when a global transcriptomic involvement is investigated. For species‐specific and cross‐species regional markers in mouse and chick pallial and striatal domains, see the Web resource: http://geserv.anat.ox.ac.uk from Belgard et al. (2013). In addition, we have proposed a new conceptual frame in evolutionary neurobiology suggesting that the cell lineage, neuronal migration, and gene expression patterns in the pallium have evolved at multiple levels of organization (Belgard and Montiel, 2013). Gene expression networks can evolve in neurons with different developmental origins, for instance, layer IV of the mammalian cerebral cortex and nidopallium in the avian brain (Fig. 6). The role of the thalamic input in inducing these coexpression modules should be further studied.

**Figure 7:**
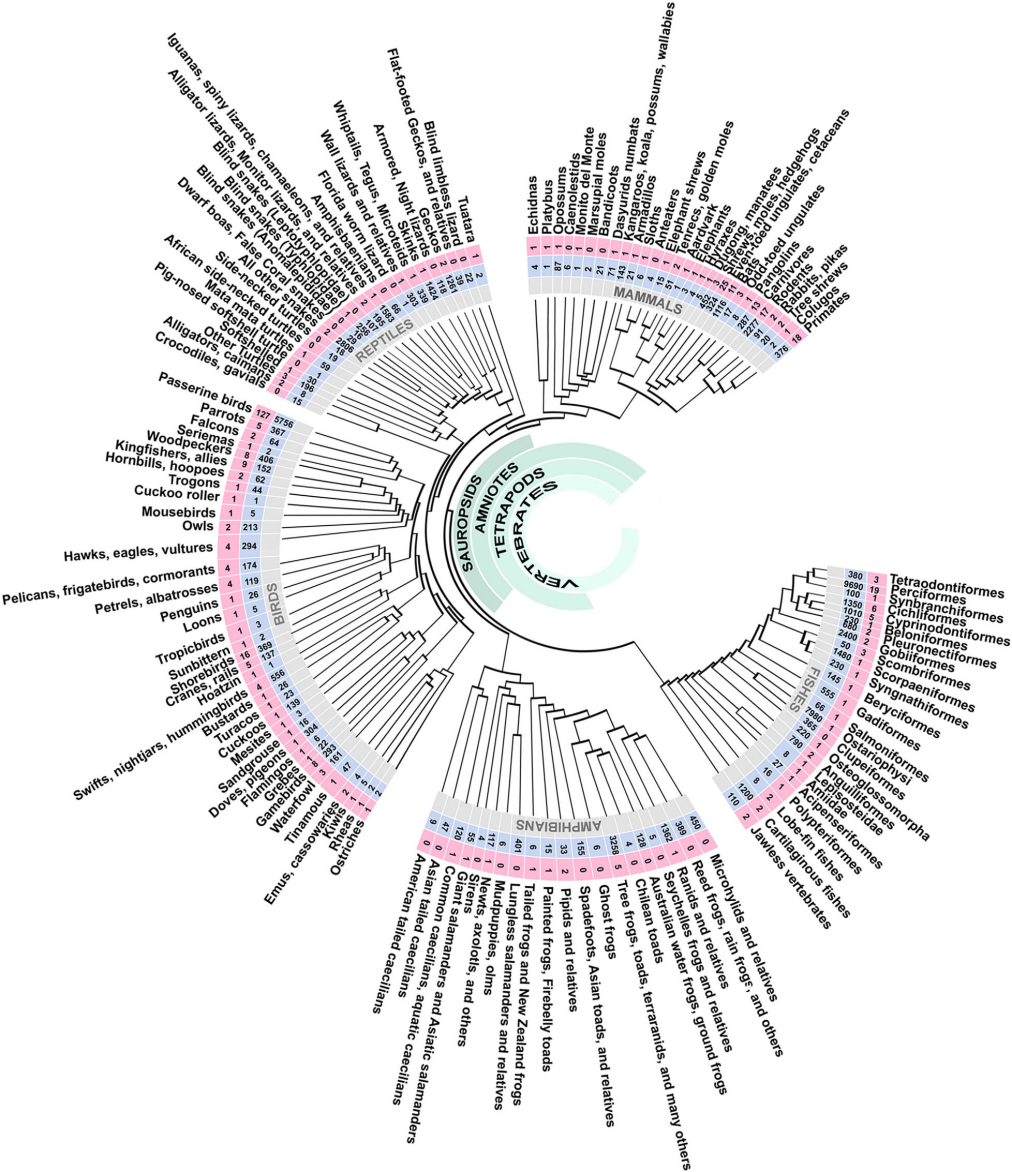
Phylogeny of the major lineages of vertebrates and their genomes availability. Numbers in light‐blue boxes represent living species and numbers in pink boxes are number of species with published and/or pending genomes. This phylogenetic tree is not time‐calibrated. More details about GenBank accession can be found at Koepfli et al. (2015; table 2) and/or https://genome10k.soe.ucsc.edu. Abbreviations in the avian brain: A, arcopallium; DP, dorsal pallium; DVR, dorsal ventricular ridge; d, dorsal tier of the mesopallium; E, entopallium; H, hyperpallium; HA, hyperpallium apicale; HAl, hyperpallium apicale lateral; HAm, hyperpallium apicale medial; HD, hyperpallium densocellulare; Hp, hippocampus; IH, intercalated hyperpallium; IHA, interstitial nucleus of hyperpallium apicale; IZ, intermediate zone; lCx, lateral cerebral cortex; LMI, lamina mesopallium intermediate; LP, lateral pallium; LS, lateral striatum; lv, lateral ventricle; MP, medial pallium; M, mesopallium; MS, medial striatum; N, nidopallium; P, pallidum; Pir, piriform cortex; ps, pial surface; S, striatum; v, ventral tier of the mesopallium; VPa, ventral pallidum; VZ, ventricular zone. Abbreviations in the mammalian brain: ACo, anterior cortical amygdalar area; Ag, basolateral amygdala; BA, basal amygdala; BST, bed nucleus of the stria terminalis; CA, central amygdala; cc, corpus callosum; CP, cortical plate; Cx, cerebral cortex; dCx, dorsal cerebral cortex; E, endopiriform complex; ec, external capsule; En, entorhinal cortex; GP, globus pallidus; Hp, hippocampus; Hyp, hypothalamus; ic, internal capsule; IZ, intermediate zone; MA, medial amygdala; MZ, Marginal zone; Pir, piriform cortex; ps, pial surface; rf, rhinal fissure; SP, subplate; SVZ, subventricular zone; Th, thalamus; VZ, ventricular zone.

More refined high‐throughput studies will allow us to track the phylogeny and ontogeny of transcriptomic signatures across species at more detailed compartmental and single cellular levels. In the coming years, the availability of numerous genome sequences through the 10k genomes project (https://genome10k.soe.ucsc.edu; Fig. 7) and other sources will open opportunities to resolve numerous outstanding issues in evolutionary neurobiology. However, all these need to be done together with the analysis of neurogenesis, migration, lineage, and functional neuronal circuit analysis.

## CONCLUSION

Comparative studies have started to reveal the differences between specific sectors of the telencephalon in vertebrates. Germinal sectors contain distinct cell types that vary in their relative proportions and arrangements in different species. These variations may be the result of differential elaboration of the germinal zone leading to the generation of more numerous and diverse neurons.

Diversification throughout evolution could result in a different processing accounting for clade‐specific cognitive performances. Cell groups could be involved in new circuits with signatures of conserved, convergent, and divergent ontogenetic histories of brain regions across vertebrates. An approach combining advances in lineage and clonal analysis with analysis of developmental and adult transcriptomes is likely to answer some of the key questions about the evolutionary origins of the mammalian neocortex. In conclusion, comparative studies have never been more exciting and important for understanding general biological features associated with the evolutionary development of our own brains and, ultimately, our behavior.

## CONFLICT OF INTEREST

No conflicts of interest to declare.

## AUTHOR CONTRIBUTIONS

All authors contributed to the writing of all parts of the review, but Juan F. Montiel mostly contributed to the transcriptomic analysis, Navneet Vasistha to the characterization of progenitors, Fernando Garcia‐Moreno to the clonal analysis parts.

## References

[cne23871-bib-0001] Abdel‐Mannan O , Cheung AFP , Molnár Z . 2008 Evolution of cortical neurogenesis. Brain Res Bull 75:398–404. 18331905 10.1016/j.brainresbull.2007.10.047

[cne23871-bib-0002] Aboitiz F , Montiel J . 2007 Origin and evolution of the vertebrate telencephalon, with special reference to the mammalian neocortex. Adv Anat Embryol Cell Biol 193:1–112. 17595827

[cne23871-bib-0003] Aboitiz F , Montiel J , Morales D , Concha M . 2002 Evolutionary divergence of the reptilian and the mammalian brains: considerations on connectivity and development. Brain Res Brain Res Rev 39:141–153. 12423764 10.1016/s0165-0173(02)00180-7

[cne23871-bib-0004] Aboitiz F , Morales D , Montiel J . 2003 The evolutionary origin of the mammalian isocortex: towards an integrated developmental and functional approach. Behav Brain Sci 26:535–552, discussion 552–585. 15179935 10.1017/s0140525x03000128

[cne23871-bib-0005] Angevine JB , Bodian D , Coulombre AJ , Edds MV Jr , Hamburger V , Jacobson M , Lyser KM , Prestige MC , Sidman RL , Varon S , Weiss PA . 1970 Embryonic vertebrate central nervous system: revised terminology. Anat Rec 166:257–261. 5414696 10.1002/ar.1091660214

[cne23871-bib-0006] Ayoub AE , Oh S , Xie Y , Leng J , Cotney J , Dominguez MH , Noonan JP , Rakic P . 2011 Transcriptional programs in transient embryonic zones of the cerebral cortex defined by high‐resolution mRNA sequencing. Proc Natl Acad Sci U S A 108:14950–14955. 21873192 10.1073/pnas.1112213108 PMC3169109

[cne23871-bib-0007] Belgard TG , Montiel JF . 2013 Things change: how comparative transcriptomics suggest the pallium has evolved at multiple levels of organization. Brain Behav Evol 82:150–152. 24081114 10.1159/000354969 PMC3881543

[cne23871-bib-0008] Belgard TG , Marques AC , Oliver PL , Abaan HO , Sirey TM , Hoerder‐Suabedissen A , García‐Moreno F , Molnár Z , Margulies EH , Ponting CP . 2011 A transcriptomic atlas of mouse neocortical layers. Neuron 71:605–616. 21867878 10.1016/j.neuron.2011.06.039 PMC3163272

[cne23871-bib-0009] Belgard TG , Montiel JF , Wang WZ , García‐Moreno F , Margulies EH , Ponting CP , Molnár Z . 2013 Adult pallium transcriptomes surprise in not reflecting predicted homologies across diverse chicken and mouse pallial sectors. Proc Natl Acad Sci U S A 110: 13150–13155. 23878249 10.1073/pnas.1307444110 PMC3740902

[cne23871-bib-0010] Betizeau M , Cortay V , Patti D , Pfister S , Gautier E , Bellemin‐Ménard A , Afanassieff M , Huissoud C , Douglas RJ , Kennedy H , Dehay C . 2013 Precursor diversity and complexity of lineage relationships in the outer subventricular zone of the primate. Neuron 80:442–457. 24139044 10.1016/j.neuron.2013.09.032

[cne23871-bib-0011] Borrell V , Calegari F . 2014 Mechanisms of brain evolution: regulation of neural progenitor cell diversity and cell cycle length. Neurosci Res 86:14–24. 24786671 10.1016/j.neures.2014.04.004

[cne23871-bib-0012] Brand S , Rakic P . 1979 Genesis of the primate neostriatum: [3H]thymidine autoradiographic analysis of the time of neuron origin in the rhesus monkey. Neuroscience 4:767–778. 113693 10.1016/0306-4522(79)90005-8

[cne23871-bib-0013] Brill MS , Ninkovic J , Winpenny E , Hodge RD , Hodge A , Ozen I , Yang R , Lepier A , Gascón S , Erdelyi F , Szabo G , Parras C , Guillemot F , Frotscher M , Berninger B , Hevner RF , Raineteau O , Götz M . 2009 Adult generation of glutamatergic olfactory bulb interneurons. Nat Neurosci 12:1524–1533. 19881504 10.1038/nn.2416 PMC2787799

[cne23871-bib-0014] Brox A , Puelles L , Ferreiro B , Medina L . 2004 Expression of the genes Emx1, Tbr1, and Eomes (Tbr2) in the telencephalon of Xenopus laevis confirms the existence of a ventral pallial division in all tetrapods. J Comp Neurol 474:562–577. 15174073 10.1002/cne.20152

[cne23871-bib-0015] Bruce LL , Neary TJ . 1995 The limbic system of tetrapods: a comparative analysis of cortical and amygdalar populations. Brain Behav Evol 46:224–234. 8564465 10.1159/000113276

[cne23871-bib-0016] Butler AB , Hodos W . 2005 Comparative vertebrate neuroanatomy. New York: Wiley‐Liss.

[cne23871-bib-0017] Charvet CJ , Owerkowicz T , Striedter GF . 2009 Phylogeny of the telencephalic subventricular zone in sauropsids: evidence for the sequential evolution of pallial and subpallial subventricular zones. Brain Behav Evol 73:285–294. 19641308 10.1159/000230673

[cne23871-bib-0018] Cheung AFP , Pollen AA , Tavare A , DeProto J , Molnár Z . 2007 Comparative aspects of cortical neurogenesis in vertebrates. J Anat 211:164–176. 17634059 10.1111/j.1469-7580.2007.00769.x PMC2375772

[cne23871-bib-0019] Cheung AFP , Kondo S , Abdel‐Mannan O , Chodroff RA , Sirey TM , Bluy LE , Webber N , DeProto J , Karlen SJ , Krubitzer L , Stolp HB , Saunders NR , Molnár Z . 2010 The subventricular zone is the developmental milestone of a 6‐layered neocortex: comparisons in metatherian and eutherian mammals. Cereb Cortex 20:1071–1081. 19726493 10.1093/cercor/bhp168

[cne23871-bib-0020] Chodroff RA , Goodstadt L , Sirey TM , Oliver PL , Davies KE , Green ED , Molnár Z , Ponting CP . 2010 Long noncoding RNA genes: conservation of sequence and brain expression among diverse amniotes. Genome Biol 11:R72. 20624288 10.1186/gb-2010-11-7-r72 PMC2926783

[cne23871-bib-0021] Clinton BK , Cunningham CL , Kriegstein AR , Noctor S , Martínez‐Cerdeño V . 2014 Radial glia in the proliferative ventricular zone of the embryonic and adult turtle, Trachemys scripta elegans. Neurogenesis 1:e970905. 10.4161/23262125.2014.970905 PMC4973586 27504470

[cne23871-bib-0022] Dehay C , Kennedy H , Kosik KS . 2015 The outer subventricular zone and primate‐specific cortical complexification. Neuron 85:683–694. 25695268 10.1016/j.neuron.2014.12.060

[cne23871-bib-0023] Del Rio JA , Soriano E , Ferrer I . 1992 Development of GABA‐immunoreactivity in the neocortex of the mouse. J Comp Neurol 326:501–526. 1484122 10.1002/cne.903260403

[cne23871-bib-0024] Dillman AA , Hauser DN , Gibbs JR , Nalls MA , McCoy MK , Rudenko IN , Galter D , Cookson MR . 2013 mRNA expression, splicing and editing in the embryonic and adult mouse cerebral cortex. Nat Neurosci 16:499–506. 23416452 10.1038/nn.3332 PMC3609882

[cne23871-bib-0025] Dugas‐Ford J , Rowell JJ , Ragsdale CW . 2012 Cell‐type homologies and the origins of the neocortex. Proc Natl Acad Sci U S A 109:16974–16979. 23027930 10.1073/pnas.1204773109 PMC3479531

[cne23871-bib-0026] Duque A , Rakic P . 2011 Different effects of bromodeoxyuridine and [3H]thymidine incorporation into DNA on cell proliferation, position, and fate. J Neurosci 31:15205–15217. 22016554 10.1523/JNEUROSCI.3092-11.2011 PMC3225276

[cne23871-bib-0027] Englund C , Fink A , Lau C , Pham D , Daza RAM , Bulfone A , Kowalczyk T , Hevner RF . 2005 Pax6, Tbr2, and Tbr1 are expressed sequentially by radial glia, intermediate progenitor cells, and postmitotic neurons in developing neocortex. J Neurosci 25:247–251. 15634788 10.1523/JNEUROSCI.2899-04.2005 PMC6725189

[cne23871-bib-0028] Fietz SA , Huttner WB . 2011 Cortical progenitor expansion, self‐renewal and neurogenesis—a polarized perspective. Curr Opin Neurobiol 21:23–35. 21036598 10.1016/j.conb.2010.10.002

[cne23871-bib-0029] Fietz SA , Lachmann R , Brandl H , Kircher M , Samusik N , Schröder R , Lakshmanaperumal N , Henry I , Vogt J , Riehn A , Distler W , Nitsch R , Enard W , Pääbo S , Huttner WB . 2012 Transcriptomes of germinal zones of human and mouse fetal neocortex suggest a role of extracellular matrix in progenitor self‐renewal. Proc Natl Acad Sci U S A 109:11836–11841. 22753484 10.1073/pnas.1209647109 PMC3406833

[cne23871-bib-0030] Flames N , Pla R , Gelman DM , Rubenstein JLR , Puelles L , Marín O . 2007 Delineation of multiple subpallial progenitor domains by the combinatorial expression of transcriptional codes. J Neurosci 27:9682–9695. 17804629 10.1523/JNEUROSCI.2750-07.2007 PMC4916652

[cne23871-bib-0031] Florio M , Huttner WB . 2014 Neural progenitors, neurogenesis and the evolution of the neocortex. Development 141:2182–2194. 24866113 10.1242/dev.090571

[cne23871-bib-0032] Fournier J , Müller CM , Laurent G . 2015 Looking for the roots of cortical sensory computation in three‐layered cortices. Curr Opin Neurobiol 31:119–126. 25291080 10.1016/j.conb.2014.09.006 PMC4898590

[cne23871-bib-0033] García‐Moreno F , Vasistha NA , Trevia N , Bourne JA , Molnár Z . 2012 Compartmentalization of cerebral cortical germinal zones in a lissencephalic primate and gyrencephalic rodent. Cereb Cortex 22:482–492. 22114081 10.1093/cercor/bhr312

[cne23871-bib-0034] Glenn Northcutt R , Kaas JH . 1995 The emergence and evolution of mammalian neocortex. Trends Neurosci 18:373–379. 7482801 10.1016/0166-2236(95)93932-n

[cne23871-bib-0035] Gray PA . 1924 The cortical lamination pattern of the opossum, Didelphys virginiana. J Comp Neurol 37:221–263.

[cne23871-bib-0036] Hansen DV , Lui JH , Parker PR , Kriegstein AR . 2010 Neurogenic radial glia in the outer subventricular zone of human neocortex. Nature 464:554–561. 20154730 10.1038/nature08845

[cne23871-bib-0037] He Z , Bammann H , Han D , Xie G , Khaitovich P . 2014 Conserved expression of lincRNA during human and macaque prefrontal cortex development and maturation. RNA 20:1103–1111. 24847104 10.1261/rna.043075.113 PMC4074677

[cne23871-bib-0038] Hoerder‐Suabedissen A . 2013 Expression profiling of mouse subplate reveals a dynamic gene network and disease association with autism and schizophrenia. Proc Natl Acad Sci U S A 110:3555–3560. 23401504 10.1073/pnas.1218510110 PMC3587197

[cne23871-bib-0039] Hoerder‐Suabedissen A , Molnár Z . 2015 Development, evolution and pathology of neocortical subplate neurons. Nat Rev Neurosci 16:133–146. 25697157 10.1038/nrn3915

[cne23871-bib-0040] Hoerder‐Suabedissen A , Wang WZ , Lee S , Davies KE , Goffinet AM , Rakic S , Parnavelas J , Reim K , Nicolic M , Paulsen O , Molnár Z . 2009 Novel markers reveal subpopulations of subplate neurons in the murine cerebral cortex. Cereb Cortex 19:1738–1750. 19008461 10.1093/cercor/bhn195

[cne23871-bib-0041] Holmgren N . 1922 Points of view concerning forebrain morphology in lower vertebrates. J Comp Neurol 34:391–459.

[cne23871-bib-0042] Hou Z‐C , Sterner KN , Romero R , Than NG , Gonzalez JM , Weckle A , Xing J , Benirschke K , Goodman M , Wildman DE . 2012 Elephant transcriptome provides insights into the evolution of eutherian placentation. Genome Biol Evol 4:713–725. 22546564 10.1093/gbe/evs045 PMC3381679

[cne23871-bib-0043] Jarvis ED , Güntürkün O , Bruce L , Csillag A , Karten H , Kuenzel W , Medina L , Paxinos G , Perkel DJ , Shimizu T , Striedter G , Wild JM , Ball GF , Dugas‐Ford J , Durand SE , Hough GE , Husband S , Kubikova L , Lee DW , Mello CV , Powers A , Siang C , Smulders TV , Wada K , White SA , Yamamoto K , Yu J , Reiner A , Butler AB , Avian Brain Nomenclature Consortium . 2005 Avian brains and a new understanding of vertebrate brain evolution. Nat Rev Neurosci 6:151–159. 15685220 10.1038/nrn1606 PMC2507884

[cne23871-bib-0044] Jarvis ED , Yu J , Rivas MV , Horita H , Feenders G , Whitney O , Jarvis SC , Jarvis ER , Kubikova L , Puck AEP , Siang Bakshi C , Martin S , McElroy M , Hara E , Howard J , Pfenning A , Mouritsen H , Chen CC , Wada K . 2013 Global view of the functional molecular organization of the avian cerebrum: mirror images and functional columns. J Comp Neurol 521:3614–3665. 23818122 10.1002/cne.23404 PMC4145244

[cne23871-bib-0045] Karten HJ . 2013 Neocortical evolution: neuronal circuits arise independently of lamination. Curr Biol 23:R12–15. 23305661 10.1016/j.cub.2012.11.013

[cne23871-bib-0046] Kelava I , Reillo I , Murayama AY , Kalinka AT , Tomancak P , Matsuzaki F , Lebrand C , Sasaki E , Schwamborn JC , Okano H , Hutner WB , Borrel V . 2012 Abundant occurrence of basal radial glia in the subventricular zone of embryonic neocortex of a lissencephalic primate, the common marmoset Callithrix jacchus. Cereb Cortex 22:469–481. 22114084 10.1093/cercor/bhr301 PMC3256412

[cne23871-bib-0047] Keyte A , Smith KK . 2012 Heterochrony in somitogenesis rate in a model marsupial, Monodelphis domestica. Evol Dev 14:93–103. 23016977 10.1111/j.1525-142X.2011.00524.x

[cne23871-bib-0048] Koepfli KP1 , Paten B ; Genome 10K Community of Scientists , O'Brien SJ . 2015 The Genome 10K Project: a way forward. Annu Rev Anim Biosci 3:57–111. 25689317 10.1146/annurev-animal-090414-014900 PMC5837290

[cne23871-bib-0049] Konopka G , Friedrich T , Davis‐Turak J , Winden K , Oldham MC , Gao F , Chen L , Wang G‐Z , Luo R , Preuss TM , Geschwind DH . 2012 Human‐specific transcriptional networks in the brain. Neuron 75:601–617. 22920253 10.1016/j.neuron.2012.05.034 PMC3645834

[cne23871-bib-0050] Krubitzer L , Kaas J . 2005 The evolution of the neocortex in mammals: how is phenotypic diversity generated? Curr Opin Neurobiol 15:444–453. 16026978 10.1016/j.conb.2005.07.003

[cne23871-bib-0051] Krupp DR , Xu PT , Thomas S , Dellinger A , Etchevers HC , Vekemans M , Gilbert JR , Speer MC , Ashley Koch AE , Gregory SG , National Birth Defects Prevention Study . 2012 Transcriptome profiling of genes involved in neural tube closure during human embryonic development using long serial analysis of gene expression (long‐SAGE). Birth Defects Res Part A Clin Mol Teratol 94:683–692. 22806986 10.1002/bdra.23040 PMC3438356

[cne23871-bib-0052] Li Z , Bammann H , Li M , Liang H , Yan Z , Phoebe Chen YP , Zhao M , Khaitovich P . 2013 Evolutionary and ontogenetic changes in RNA editing in human, chimpanzee, and macaque brains. RNA 19:1693–1702. 24152549 10.1261/rna.039206.113 PMC3884655

[cne23871-bib-0053] Liu X , Somel M , Tang L , Yan Z , Jiang X , Guo S , Yuan Y , He L , Oleksiak A , Zhang Y , Li N , Hu Y , Chen W , Qiu Z , Pääbo S , Khaitovich P . 2012 Extension of cortical synaptic development distinguishes humans from chimpanzees and macaques. Genome Res 22:611–622. 22300767 10.1101/gr.127324.111 PMC3317144

[cne23871-bib-0054] Lui JH , Hansen DV , Kriegstein AR . 2011 Development and evolution of the human neocortex. Cell 146:18–36. 21729779 10.1016/j.cell.2011.06.030 PMC3610574

[cne23871-bib-0055] Manger PR , Slutsky DA , Molnár Z . 2002 Visual subdivisions of the dorsal ventricular ridge of the iguana (Iguana iguana) as determined by electrophysiologic mapping. J Comp Neurol 453:226–246. 12378585 10.1002/cne.10373

[cne23871-bib-0056] Marín O , Müller U . 2014 Lineage origins of GABAergic versus glutamatergic neurons in the neocortex. Curr Opin Neurobiol 26:132–141. 24549207 10.1016/j.conb.2014.01.015 PMC4159607

[cne23871-bib-0057] Martínez‐Cerdeño V , Noctor SC , Kriegstein AR . 2006 The role of intermediate progenitor cells in the evolutionary expansion of the cerebral cortex. Cereb Cortex 16(Suppl 1):i152–161. 16766701 10.1093/cercor/bhk017

[cne23871-bib-0058] Medina L , Abellán A . 2009 Development and evolution of the pallium. Semin Cell Dev Biol 20:698–711. 19393324 10.1016/j.semcdb.2009.04.008

[cne23871-bib-0059] Medina L , Reiner A . 2000 Do birds possess homologues of mammalian primary visual, somatosensory and motor cortices? Trends Neurosci 23:1–12. 10631781 10.1016/s0166-2236(99)01486-1

[cne23871-bib-0060] Miller JA , Horvath S , Geschwind DH . 2010 Divergence of human and mouse brain transcriptome highlights Alzheimer disease pathways. Proc Natl Acad Sci U S A 107:12698–12703. 20616000 10.1073/pnas.0914257107 PMC2906579

[cne23871-bib-0061] Miller JA , Ding SL , Sunkin SM , Smith KA , Ng L , Szafer A , Ebbert A , Riley ZL , Royall JJ , Aiona K , Arnold JM , Bennet C , Bertagnolli D , Brouner K , Butler S , Caldejon S , Carey A , Cuhaciyan C , Dalley RA , Dee N , Dolbeare TA , Facer BA , Feng D , Fliss TP , Gee G , Goldy J , Gourley L , Gregor BW , Gu G , Howard RE , Jochim JM , Kuan CL , Lau C , Lee CK , Lee F , Lemon TA , Lesnar P , McMurray B , Mastan N , Mosqueda N , Naluai‐Cecchini T , Ngo NK , Nyhus J , Oldre A , Olson E , Parente J , Parker PD , Parry SE , Stevens A , Pletikos M , Reding M , Roll K , Sandman D , Sarreal M , Shapouri S , Shapovalova NV , Shen EH , Sjoquist N , Slaughterbeck CR , Smith M , Sodt AJ , Williams D , Zöllei L , Fischl B , Gerstein MB , Geschwind DH , Glass IA , Hawrylycz MJ , Hevner RF , Huang H , Jones AR , Knowles JA , Levitt P , Phillips JW , Sestan N , Wohnoutka P , Dang C , Bernard A , Hohmann JG , Lein ES . 2014 Transcriptional landscape of the prenatal human brain. Nature 508:199–206. 24695229 10.1038/nature13185 PMC4105188

[cne23871-bib-0062] Molnár Z . 2011 Evolution of cerebral cortical development. Brain Behav Evol 78:94–107. 21691047 10.1159/000327325

[cne23871-bib-0063] Molnár Z , Butler AB . 2002 The corticostriatal junction: a crucial region for forebrain development and evolution. Bioessays 6:530–541 12111736 10.1002/bies.10100

[cne23871-bib-0064] Molnár Z , Pollen A . 2014 How unique is the human neocortex? Development 141:11–16. 24346696 10.1242/dev.101279

[cne23871-bib-0065] Molnár Z , Métin C , Stoykova A , Tarabykin V , Price DJ , Francis F , Meyer G , Dehay C , Kennedy H . 2006 Comparative aspects of cerebral cortical development. Eur J Neurosci 23:921–934. 16519657 10.1111/j.1460-9568.2006.04611.x PMC1931431

[cne23871-bib-0066] Montiel JF , Molnár Z . 2013 The impact of gene expression analysis on evolving views on avian brain organization. J Comp Neurol 521:3604–3613. 23818089 10.1002/cne.23403

[cne23871-bib-0067] Montiel JF , Wang WZ , Oeschger FM , Hoerder‐Suabedissen A , Tung WL , García‐Moreno F , Holm IE , Villalón A , Molnár Z . 2011 Hypothesis on the dual origin of the Mammalian subplate. Front Neuroanat 5:25. 21519390 10.3389/fnana.2011.00025 PMC3078748

[cne23871-bib-0068] Moreno N , González A . 2007 Evolution of the amygdaloid complex in vertebrates, with special reference to the anamnio‐amniotic transition. J Anat 211:151–163. 17634058 10.1111/j.1469-7580.2007.00780.x PMC2375767

[cne23871-bib-0069] Nomura T , Takahashi M , Hara Y , Osumi N . 2008 Patterns of neurogenesis and amplitude of Reelin expression are essential for making a mammalian‐type cortex. PLoS ONE 3:e1454. 18197264 10.1371/journal.pone.0001454 PMC2175532

[cne23871-bib-0070] Nomura T , Gotoh H , Ono K . 2013 Changes in the regulation of cortical neurogenesis contribute to encephalization during amniote brain evolution. Nat Commun 4:2206. 23884180 10.1038/ncomms3206

[cne23871-bib-0071] Northcutt RG . 2003 The use and abuse of developmental data. Behav Brain Sci 26:565–566.

[cne23871-bib-0072] Oeschger FM , Wang WZ , Lee S , García‐Moreno F , Goffinet AM , Arbonés ML , Rakic S , Molnár Z . 2012 Gene expression analysis of the embryonic subplate. Cereb Cortex 22:1343–1359. 21862448 10.1093/cercor/bhr197 PMC4972418

[cne23871-bib-0073] Pfenning AR , Hara E , Whitney O , Rivas MV , Wang R , Roulhac PL , Howard JT , Wirthlin M , Lovell PV , Ganapathy G , Mouncastle J , Moseley MA , Thompson JW , Soderblom EJ , Iriki A , Kato M , Gilbert MT , Zhang G , Bakken T , Bongaarts A , Bernard A , Lein E , Mello CV , Hartemink AJ , Jarvis ED . 2014 Convergent transcriptional specializations in the brains of humans and song‐learning birds. Science 346:1256846. 25504733 10.1126/science.1256846 PMC4385736

[cne23871-bib-0074] Puelles L . 1995 A segmental morphological paradigm for understanding vertebrate forebrains. Brain Behav Evol 46:319–337. 8564469 10.1159/000113282

[cne23871-bib-0075] Puelles L , Kuwana E , Puelles E , Bulfone A , Shimamura K , Keleher J , Smiga S , Rubenstein JLR . 2000 Pallial and subpallial derivatives in the embryonic chick and mouse telencephalon, traced by the expression of the genes Dlx‐2, Emx‐1, Nkx‐2.1, Pax‐6, and Tbr‐1. J Comp Neurol 424:409–438. 10906711 10.1002/1096-9861(20000828)424:3<409::aid-cne3>3.0.co;2-7

[cne23871-bib-0076] Puzzolo E , Mallamaci A . 2010 Cortico‐cerebral histogenesis in the opossum Monodelphis domestica: generation of a hexalaminar neocortex in the absence of a basal proliferative compartment. Neural Dev 5:8. 20302607 10.1186/1749-8104-5-8 PMC2859365

[cne23871-bib-0077] Reillo I , Borrell V . 2012 Germinal zones in the developing cerebral cortex of ferret: ontogeny, cell cycle kinetics, and diversity of progenitors. Cereb Cortex 22:2039–2054. 21988826 10.1093/cercor/bhr284

[cne23871-bib-0078] Reillo I , de Juan Romero C , Garcia‐Cabezas MA , Borrell V . 2011 A role for intermediate radial glia in the tangential expansion of the mammalian cerebral cortex. Cereb Cortex 21:1674–1694. 21127018 10.1093/cercor/bhq238

[cne23871-bib-0079] Reilly SK , Yin J , Ayoub AE , Emera D , Leng J , Cotney J , Sarro R , Rakic P , Noonan JP . 2015 Evolutionary genomics. Evolutionary changes in promoter and enhancer activity during human corticogenesis. Science 347:1155–1159. 25745175 10.1126/science.1260943 PMC4426903

[cne23871-bib-0080] Reiner A . 1991 A comparison of neurotransmitter‐specific and neuropeptide‐specific neuronal cell types present in the dorsal cortex in turtles with those present in the isocortex in mammals: implications for the evolution of isocortex. Brain Behav Evol 38:53–91. 1683805 10.1159/000114379

[cne23871-bib-0081] Reiner AJ . 2000 A hypothesis as to the organization of cerebral cortex in the common amniote ancestor of modern reptiles and mammals. Novartis Found Symp 228:83–102, discussion 102–113. 10929318 10.1002/0470846631.ch7

[cne23871-bib-0082] Rowell JJ , Ragsdale CW . 2012 BrdU birth dating can produce errors in cell fate specification in chick brain development. J Histochem Cytochem 60:801–810. 22859704 10.1369/0022155412458588 PMC3524571

[cne23871-bib-0083] Sessa A , Mao C‐A , Chai, Colasante G , Nini A , Klein WH . 2010 Tbr2‐positive intermediate (basal) neuronal progenitors safeguard cerebral cortex expansion by controlling amplification of pallial glutamatergic neurons and attraction of subpallial GABAergic interneurons. J Comp Neurol 24:1816–1826. 10.1101/gad.575410 PMC2922508 20713522

[cne23871-bib-0084] Shitamukai A , Konno D , Matsuzaki F . 2011 Oblique radial glial divisions in the developing mouse neocortex induce self‐renewing progenitors outside the germinal zone that resemble primate outer subventricular zone progenitors. J Neurosci 31:3683–3695. 21389223 10.1523/JNEUROSCI.4773-10.2011 PMC6622781

[cne23871-bib-0085] Smart IHM , Dehay C , Giroud P , Berland M , Kennedy H . 2002 Unique morphological features of the proliferative zones and postmitotic compartments of the neural epithelium giving rise to striate and extrastriate cortex in the monkey. Cereb Cortex 12:37–53. 11734531 10.1093/cercor/12.1.37 PMC1931430

[cne23871-bib-0086] Striedter GF , Keefer BP . 2000 Cell migration and aggregation in the developing telencephalon: pulse‐labeling chick embryos with bromodeoxyuridine. J Neurosci 20:8021–8030. 11050123 10.1523/JNEUROSCI.20-21-08021.2000 PMC6772730

[cne23871-bib-0087] Striedter GF , Northcutt RG . 1991 Biological hierarchies and the concept of homology. Brain Behav Evol 38:177–189. 1663811 10.1159/000114387

[cne23871-bib-0088] Stubbs D , DeProto J , Nie K , Englund C , Mahmud I , Hevner R , Molnár Z . 2009 Neurovascular congruence during cerebral cortical development. Cereb Cortex 19:i32–i41. 19386634 10.1093/cercor/bhp040 PMC2693536

[cne23871-bib-0089] Suzuki IK , Kawasaki T , Gojobori T , Hirata T . 2012 The temporal sequence of the mammalian neocortical neurogenetic program drives mediolateral pattern in the chick pallium. Dev Cell 22:863–870. 22424929 10.1016/j.devcel.2012.01.004

[cne23871-bib-0090] Tarabykin V , Stoykova A , Usman N , Gruss P . 2001 Cortical upper layer neurons derive from the subventricular zone as indicated by Svet1 gene expression. Development 128:1983–1993. 11493521 10.1242/dev.128.11.1983

[cne23871-bib-0091] Taverna E , Götz M , Huttner WB . 2014 The cell biology of neurogenesis: toward an understanding of the development and evolution of the neocortex. Annu Rev Cell Dev Biol 30:465–502. 25000993 10.1146/annurev-cellbio-101011-155801

[cne23871-bib-0092] Tsai HM , Garber BB , Larramendi LM . 1981 3H‐thymidine autoradiographic analysis of telencephalic histogenesis in the chick embryo: I. Neuronal birthdates of telencephalic compartments in situ. J Comp Neurol 198:275–292. 7240446 10.1002/cne.901980207

[cne23871-bib-0093] Vasistha NA , García‐Moreno F , Arora S , Cheung AFP , Arnold SJ , Robertson EJ , Molnár Z . 2014 Cortical and clonal contribution of Tbr2 expressing progenitors in the developing mouse brain. Cereb Cortex pii: bhu125. 10.1093/cercor/bhu125 PMC4585488 24927931

[cne23871-bib-0094] Wang Y , Brzozowska‐Prechtl A , Karten HJ . 2010 Laminar and columnar auditory cortex in avian brain. Proc Natl Acad Sci U S A 107:12676–12681. 20616034 10.1073/pnas.1006645107 PMC2906560

[cne23871-bib-0095] Wang WZ , Oeschger FM , Montiel JF , García‐Moreno F , Hoerder‐Suabedissen A , Krubitzer L , Ek CJ , Saunders NR , Reim K , Villalón A , Molnár Z . 2011a Comparative aspects of subplate zone studied with gene expression in sauropsids and mammals. Cereb Cortex 21:2187–2203. 21368089 10.1093/cercor/bhq278

[cne23871-bib-0096] Wang X , Tsai J‐W , LaMonica B , Kriegstein AR . 2011b A new subtype of progenitor cell in the mouse embryonic neocortex. Nat Neurosci 14:555–561. 21478886 10.1038/nn.2807 PMC3083489

[cne23871-bib-0097] Zelenitsky DK , Therrien F , Ridgely RC , McGee AR , Witmer LM . 2011 Evolution of olfaction in non‐avian theropod dinosaurs and birds. Proc Biol Sci 278:3625–3634. 21490022 10.1098/rspb.2011.0238 PMC3203493

